# Implementing an Injury Prevention Briefing to aid delivery of key fire safety messages in UK children’s centres: qualitative study nested within a multi-centre randomised controlled trial

**DOI:** 10.1186/1471-2458-14-1256

**Published:** 2014-12-10

**Authors:** Kate Beckett, Trudy Goodenough, Toity Deave, Sally Jaeckle, Lisa McDaid, Penny Benford, Mike Hayes, Elizabeth Towner, Denise Kendrick, Keeping Children Safe Programme study group

**Affiliations:** Centre for Child & Adolescent Health, Faculty of Health and Life Sciences, University of the West of England, Bristol, UK; Early Years Learning, Bristol City Council, City Hall, College Green, Bristol, UK; NHS Clinical Research and Trials Unit, Norwich Medical School, Norfolk and Norwich University Hospital, University of East Anglia, Norwich, UK; School of Medicine, Division of Primary Care, University of Nottingham, University Park, Nottingham, UK; Child Accident Prevention Trust, Canterbury Court, 1-3 Brixton Road, London, UK

**Keywords:** Fire safety, Injury prevention, Children’s centre, Context, Intervention, Implementation, Facilitation

## Abstract

**Background:**

To improve the translation of public health evidence into practice, there is a need to increase practitioner involvement in initiative development, to place greater emphasis on contextual knowledge, and to address intervention processes and outcomes. Evidence that demonstrates the need to reduce childhood fire-related injuries is compelling but its translation into practice is inconsistent and limited. With this knowledge the Keeping Children Safe programme developed an "Injury Prevention Briefing (IPB)" using a 7 step process to combine scientific evidence with practitioner contextual knowledge. The IPB was designed specifically for children’s centres (CCs) to support delivery of key fire safety messages to parents. This paper reports the findings of a nested qualitative study within a clustered randomised controlled trial of the IPB, in which staff described their experiences of IPB implementation to aid understanding of why or how the intervention worked.

**Methods:**

Interviews were conducted with key staff at 24 CCs participating in the two intervention arms: 1) IPB supplemented by initial training and regular facilitation; 2) IPB sent by post with no facilitation. Framework Analysis was applied to these interview data to explore intervention adherence including; exposure or dose; quality of delivery; participant responsiveness; programme differentiation; and staff experience of IPB implementation. This included barriers, facilitators and suggested improvements.

**Results:**

83% of CCs regarded the IPB as a simple, accessible tool which raised awareness, and stimulated discussion and behaviour change. 15 CCs suggested minor modifications to format and content. Four levels of implementation were identified according to content, frequency, duration and coverage. Most CCs (75%) achieved ‘extended’ or ‘essential’ IPB implementation. Three universal factors affected all CCs: organisational change and resourcing; working with hard to engage groups; additional demands of participating in a research study. Six specific factors were associated with the implementation level achieved: staff engagement and training; staff continuity; adaptability and flexibility; other agency support; conflicting priorities; facilitation. CCs achieving high implementation levels increased from 58% (no facilitation) to 92% with facilitation.

**Conclusion:**

Incorporating service provider perspectives and scientific evidence into health education initiatives enhances potential for successful implementation, particularly when supplemented by ongoing training and facilitation.

**Electronic supplementary material:**

The online version of this article (doi:10.1186/1471-2458-14-1256) contains supplementary material, which is available to authorized users.

## Background

Factors that impede the translation of scientific evidence into practice are a recurring theme in the literature [1–5]. Implementation has been described as the ‘the Achilles’ heel of innovation’ [3] and is a major issue for public health [1, 4]. Despite an expanding body of robust scientific evidence and political enthusiasm for best practice in health promotion, the uptake of evidence into practice is rarely straightforward [1–5]. Contributory factors are: lack of academic emphasis on process rather than outcome, lack of practitioner involvement or contextual knowledge and centrally driven priorities [1–3]. In the case of childhood thermal injury, transforming evidence into effective interventions among at-risk populations has had limited success [6–8]. In this case, the synthesis of art and science, through combining systematic evidence of effective interventions with practitioner knowledge of context, may have greater potential to effect change [1].

Evidence highlighting the need for effective interventions to reduce UK childhood fire-related injuries (any injury occurring during a house fire, either from flames or the associated smoke) is compelling [1, 8–10]. These types of injury are a major public health concern; [1, 8–10] they have the steepest social gradient of all injuries [9, 11] and enduring implications for child, family, the National Health Service (NHS) and society [9, 11–13]. Patterns of fire-related injury are closely linked to children’s age and developmental stages [7, 9, 10] and most are potentially preventable [7–9]. Parental education is therefore important [1, 9]. Successive government policies and NHS directives have emphasised the importance of child injury reduction, especially through targeting at-risk families [9, 10, 14]. In the UK children’s centres (CCs) working in the most disadvantaged areas have a pivotal role in delivering health education to these groups and run a range of health promotion programmes [9]. However, there is little evidence of a consistent, systematic evidence-based approach to development, implementation and monitoring of fire safety interventions in this environment where there are unique organisational and audience related challenges to delivery [6, 9, 15, 16] and interventions designed for other contexts may not work [1, 2].

The Keeping Children Safe at Home (KCS) [9] programme is a five year multi-centre research programme involving a series of interlinked studies aimed at developing a better understanding of unintentional injury prevention in pre-school children. This paper relates to one component of this programme: development and trial of an evidence-based fire safety guidance document, referred to as an Injury Prevention Briefing (IPB), specifically for use in CCs [1]. The IPB was developed using an innovative seven step process (based on Kelly et al. [17] and described in full in Brussoni et al. [1]) to combine scientific evidence of what works, or can be regarded as best practice, with the practical experience of people who already run health education programmes in the field. Systematic reviews and meta-analyses conducted within the NIHR-funded KCS programme contributed evidence to this process [9]. The resulting IPB is a toolkit providing guidance, information and activities to aid delivery of five key fire safety messages by CC staff. The KCS team proposed that this approach could improve implementation in real world settings [1, 9]. To test this hypothesis a pragmatic, multi-centred cluster randomised controlled trial with nested qualitative study [9] was conducted in 36 recruited CCs in the 4 UK KCS study sites (Bristol, Newcastle, Norwich and Nottingham). These 36 CCs (nine per study site) were randomly allocated to one of the three trial arms which aimed to contrast effects of: 1) IPB delivery supplemented by initial training and on-going facilitation (IPB+); 2) IPB mailed to CC (IPB only); 3) usual CC fire safety activity (control). The primary outcome measure for the trial was the proportion of families with a fire escape plan 12 months after the intervention commenced. Trial methods including sample selection criteria are described in detail in the KCS published protocol [9].

However this paper reports on the nested qualitative study in which staff from the 24 CCs (in the intervention arms) described their experience of delivering the key safety messages in the IPB. This methodology was explicitly chosen to gain insight into practitioner perspectives on IPB implementation. The interview schedule and analysis was guided by Carroll et al’s [18] ‘Implementation Fidelity Framework’, a validated tool for testing implementation efficacy of a variety of interventions in diverse settings. This conceptual framework supports measurement of ‘adherence’ to an interventions predefined components (content, coverage, frequency and duration) and ‘moderators’ affecting the delivery process (intervention complexity, facilitation strategies, quality of delivery and participant response). Implementation fidelity is according to Carroll et al. [18] extremely difficult to achieve; the relationship between an intervention and its desired outcomes is frequently ‘moderated’ by other factors. This framework provides a structure within which to answer the following research questions addressed by this paper:

-  How many of the 24 CCs in the intervention arms achieved implementation fidelity?
-  What level of IPB implementation did CCs achieve?
-  What factors influenced implementation?
-  Did the IPB facilitate implementation in this context?
-  What improvements can be made to the IPB?

## Methods

The 24 participating CCs in the two intervention arms were asked to deliver key fire safety messages in the IPB over a 12 month period to parents who had been recruited to the trial and completed consent forms and baseline questionnaires. Differences between IPB only and IPB+ intervention arms are described in full in Table 1.

**Table 1:** **Differences between IPB only and IPB+ intervention arms**

IPB only	IPB+
• Received the IPB document in the post	• Received the IPB and a training session covering IPB content and use delivered by the Child Accident Prevention Trust and KCS research team
• Asked to use it as they would any other information	• Key content: Expected to deliver at least one session to participating families based on five key IPB fire prevention messages:
	▪ Importance of smoke alarms
	▪ Having a fire escape plan
	▪ Causes of house fires
	▪ Children’s behaviour in a fire
	▪ Following a bed time routine
	• If unable to cover all five messages directed to focus on the following two essential ones: importance of smoke alarms and fire escape plans.
	• KCS researcher facilitation contacts took place at one, three and eight months. These took the form of an interview about progress and discussion of alternative strategies and approaches.

CCs in both arms completed activity logs and participated in a semi-structured audio-recorded interview at the end of the 12 month intervention period. Interviews designed to explore and measure the ‘implementation and fidelity’ of IPB delivery were preceded by an online questionnaire with brief answers (yes/no) to inform the dialogue. Participants were assured of anonymity and confidentiality both for themselves and their organisation, ethical approval and written informed consent were obtained prior to interview. Interviews were undertaken face–to-face by KCS researchers in a quiet location with only interviewer and interviewee(s) present. The interview covered the four elements of ‘adherence’ and four implementation ‘moderators’ outlined in the Implementation Fidelity Framework [18]. It also contained more open questions about CC staff experience of IPB implementation including barriers, facilitators and suggested improvements (see Appendix 1 for example questions). All interviews were transcribed verbatim by non-KCS staff at one site.

Initial analysis was conducted by KB and TG using Framework Analysis [19] supported by the QSR NVivo 10 software package. This methodology was chosen to permit structured analysis of a priori themes (derived from the Implementation Fidelity Framework) and exploration of additional themes that emerged from the data [9, 19]. Analysis followed the sequence outlined in detail in Gale et al. [19] and included both: 1) systematic coding to reflect the structure of the interview questions and 2) open coding to capture additional or hidden meaning and anomalous data. KB and TG independently coded 3 transcripts each and developed an analytical framework through further cycles of coding. Assigned codes were discussed to ensure discrepancies and disagreements were identified and addressed and grouped into meaningful categories. This initial coding framework was reviewed by the trial chief investigator (DK) and two senior researchers (TD and ET) and subsequently applied to the remaining transcripts. Codes and categories assigned were iteratively discussed and refined to identify broader themes and generate a coding matrix to assist in identifying relationships and patterns in the data.

Four "implementation levels" were devised from this matrix to reflect the four elements of ‘adherence’ in the Implementation Fidelity Framework (content, coverage, frequency and duration of delivery) in relation to the IPB [18]. The data were subsequently classified according to the level of implementation achieved by each CC. Any disagreement was resolved through discussion between TG and KB or referral to senior researchers (ET, TD). CCs classification was then reviewed by researchers from all four study sites based on their local knowledge of IPB implementation. Where necessary the interview data were verified against other sources of data such as trial activity logs. This was particularly important where CC staff changes had occurred between trial inception and completion and the interviewee lacked knowledge of preceding stages. This verification resulted in reclassification of three cases. Finally, relationships between implementation level and other variables (moderators) apparent in the data were explored to determine barriers and facilitators affecting IPB implementation.

### Ethics statement

Ethical approval for the study was provided by East Midlands - Derby Research Ethics Committee, of the National Research Ethics Service, NHS Health Research Authority. Participant’s written informed consent included the statement: ‘I agree that anonymous direct quotes from the contact may be used in the study reports’.

## Results

Twenty-four interviews were conducted with managers and/or staff responsible for IPB delivery. This included (11 Centre Co-ordinators or Managers; 11 Practitioners (Family Support Workers, Outreach Workers and Early Years Practitioners) and 2 Health Visitors). Staff from all 24 CCs participated in the 12 month interview, comprising three CCs in the IPB+ and three in the IPB only arm per study site; no-one chose not to participate.

### Numbers of children’s centres achieving implementation fidelity

Eighteen (75%) CCs achieved ‘high implementation fidelity’ according to Carroll’s framework [18] by adhering to the IPB in terms of implementation content, frequency, duration, and coverage. Six CCs did not meet the criteria for IPB implementation (defined in the following section).

### Levels of implementation achieved

Analysis of the data suggests four distinct levels of IPB implementation that were associated with different styles of delivery (summarised below).

-  ‘Extended’ implementation included diverse delivery methods, wide coverage, key messages and content including additional information not presented in the IPB.
-  ‘Essential’ implementation - used minimum delivery methods and key content only (as described in the ‘The differences between IPB only and IPB+ intervention arms’ section).
-  ‘Minimal’ implementation - some recorded attempt at IPB related activity but insufficient to fulfil ‘essential implementation’ criteria.
-  ‘No implementation’ - CCs who did not implement any aspect of the IPB.

More detailed classification criteria and numbers of CCs in each group are provided in Table 2.

**Table 2:** **Classification criteria for levels of implementation (by numbers of CCs)**

Level	Extended	Essential	Minimal	Non-implementation
**Criteria**	• ≥ 2 delivery methods (e.g. group sessions, display boards, postal information, home visits, specific events)	• Delivered via at least one group session	• Recorded attempt at IPB related activity but insufficient to fulfil ‘Essential implementation’ criteria	• No evidence of any IPB related activity although may have provided usual fire safety activity
	• ≥2 messages^1^	• 2 messages^1^		
	• Fully integrated into existing CCs health promotion activity	• Discrete delivery or limited integration into other CCs sessions		
	• Active engagement with wide population of parents (beyond trial participants)	• Engaged with trial parents and/or passive involvement of wider community		
	• Use of IPB and additional information or content	• Used IPB information		
	• Delivered to more than 1 group	• Delivery to one group of parents		
**No. of CCs**	**10**	**8**	**5**	**1**

^1^including ‘Importance of smoke alarms’ (SA) and ‘Fire escape planning’ (FEP).

During the trial period, many CCs participated in fire safety activities unrelated to the IPB and these were also documented and noted within the interview. These four different implementation levels were captured in the participant quotes in Table 3.

**Table 3:** **The four implementation levels as described in participant accounts**

Level	Participant description
**Extended**	*‘We had boards and things up … we put up photographs after the events…. The fire safety in the home booklet pages photocopied out of books and stuck up …and we … encouraged people to come along to the workshops … slotting activities into a session, that’s already running … we’ve tried to do it …. in different ways … we set up the large training room with … hair straighteners on the floor … to try and make it a bit more interactive ….we watched the fancy a cuppa DVD which is from the child action prevention people… So we tried doing a lot of different things to make it a bit more interesting.’* (Site B: IPB+)
**Essential**	*‘Initially we’d looked through the … IPB, to see exactly what it entailed … and decided that … we’d try and construct a lesson plan to give possibly two sessions at that point because I was obviously time constrained…it was just myself and the student social worker that provided the information … we just had to adapt to make the timings a lot shorter.’* (Site A: IPB+)
**Minimal**	*‘We’d made… it wasn’t … official as such but we talked about how we were going to use it so we’d said that we’d would run workshops but … after contacting all the parents and not having the interest we didn’t go ahead if we had had the interest we would have gone ahead and made proper plans.’* (Site C: IPB only)
**Non-implementation**	*‘We never had the briefing the injury prevention briefing book so … I think right from the beginning we have been at a misunderstanding. Also our manager at that time, two of the staff that set it up have left, our strategic manager … has also been on long term sick, the manager of our team has retired and we are going through a management of change so we have been short staffed… I don’t really know what IPB is.’* (Site C: IPB only)

### Factors influencing IPB implementation

Framework analysis enabled us to explore the fit between our data and the four implementation moderators defined by Carroll et al. [18]. However, it also suggested important differences in the effect exerted by different types of delivery moderator; in particular between: 1) ‘universal moderators’ which influenced the strength of implementation in all CCs but did not distinguish between implementation levels or, 2) ‘specific moderators’ which affected some CCs only and had a direct impact on implementation levels achieved. In addition, some moderators defined in the Implementation Fidelity Framework e.g. participant responsiveness, appeared too broad to capture the range of factors influencing CC staff and parental engagement and response. Moderators affecting delivery also acted differently to those inherent in the intervention itself. Consequently, while our analysis was guided by the Implementation Fidelity Framework [18] the following results are organised to reflect these alternative patterns and relationships (Table 4 maps our findings to this work).

**Table 4:** **Mapping the relationship between the 4 implementation moderators defined in the implementation Fidelity Framework (IFF) and KCS study ‘Universal’ and ‘Specific’ moderators**

***KCS Moderators***	***Universal Moderators***		***Specific Moderators***
**IFF Moderators**	*Delivery factors*	*Intervention factors*	*Delivery factors*
**4. Intervention Complexity**	KCS trial processes	IPB Complexity	
**5. Facilitation Strategies**			KCS facilitation
			External Agency Support
**6. Quality of Delivery**			Adaptability and flexibility
**7. Participant Responsiveness**	Organisational change, time and resources		Staff engagement and training
	Working with hard to engage groups		Staff continuity
			Conflicting Priorities

1. Universal delivery moderators

There were three universal moderators of IPB implementation: 1) organisational change, time and resources; 2) working with hard to engage groups; 3) engaging with KCS study processes. These moderators affected IPB delivery but were not related to the intervention itself.

- Organisational change, time and resources:

All participants described major current, imminent or recent restructuring which made it hard to deliver services and implement health promotion messages, including the IPB. CCs were subject to constant change in management, staffing and budgets. Uncertainty about future developments permeated all accounts and even those who were experiencing relative stability anticipated forthcoming re-organisation.

*‘I don’t think we have used it to the extent that I would have liked but I think unfortunately … this has been a time of some huge change.’* (Site C: IPB+)

*‘The local authority …put out to tender the children’s centres and so we’ve been waiting … to see who… is going to be successful in the bid … it brings a lot of unrest to workers …so we’re in a great period of change’.* (Site A: IPB only)

All CCs described significant resource constraints, an expanding or changing remit and pressing priorities which made it difficult to take on new projects.

*‘Some staff have been made redundant … some of them haven’t actually left yet but they’ve been given a redundancy notice … some have had to a cover for staff who’ve left’.* (Site B: IPB+)

‘*How do you deliver things with the time constraints that we have … with heavy safeguarding cases and heavy caseload in general*’. (Site D: IPB only)

While organisational change, in general, was not a good indicator of the level of implementation, one specific aspect, namely ‘staff consistency’ appeared to have a pivotal effect. This is discussed in section 2.

b) Working with hard to engage groups:

All CCs described issues which they perceived as affecting their parent population’s engagement, including competing or more urgent issues or life changes:

‘*A lot of the families have chaotic lives, … they fully intend to at the time of the saying yes I will, but then something happens and they’ve got another priority to deal with.’* (Site B: IPB only)

*‘Not to say that fire safety isn’t a massive priority … but if you walk into a family home and they’ve not got any money, the bank account has been shut or they’ve not got any food … they’ve had a letter from children’s services … you need to deal with that on that day’.* (Site D: IPB+)

There were also policy changes which CC staff felt impacted directly on CC parents:

*‘The amount of changes that there have been within the county in terms of … like hardship fund, council changes, we’ve had to deal with all of those things with our families which have been their priority.’* (Site B IPB+)

CC staff felt that haphazard attendance and transient lifestyle was an additional barrier to parental engagement:

*‘Attendance at groups and things is so hit and miss that I don’t think it’s a reflection on what the activity was … I just think it’s … the nature of the game really.’* (Site D IPB+)

*‘The biggest battle for us is because you know with our stay and play group parents are never consistent … that has been the biggest stumbling block …with us as well our families move around so a lot of the families … have either moved on or we are actually no longer working with so we don’t have that contact with them anymore.’* (Site C: IPB only)

Even when parents did attend there were factors which CC staff felt impacted on their engagement such as communication difficulties, peer pressure, mismatch between parents’ expectations and CCs’ remit, childcare issues, perceived relevance of topics, parental perception that they already knew about fire safety and/or fear that their knowledge could be found lacking:

*‘They … feel like you’re putting them on the spot for answers and …* (being tested) *… that’s probably not an enjoyable experience.’* (Site D: IPB+)

c) KCS trial processes:

Trial processes and timings introduced complex additional demands on all participating CCs and added to the complexity of the intervention. In particular, the requirement to provide sessions to recruited parents conflicted with usual provision of a more flexible programme:

*‘I think that’s a side effect of it having been a study … we obviously wanted the 30 parents that we could ….follow up 12 months later … you had to focus it… I suppose in a more naturalistic situation which …this wasn’t … you would do more.’* (Site A: IPB+)

This somewhat artificial component was an impediment for some but appeared to facilitate limited implementation in two CCs where trial structure and expectations spurred staff on to deliver discrete targeted sessions. Higher levels of IPB implementation were frequently achieved by setting aside study expectations to deliver to recruited parents and opportunistically delivering messages to all attendees.

These three ‘universal moderators’ impacted on all CCs’ implementation but did not have a clear relationship with implementation level. However, the following six ‘specific moderators’ affecting delivery were strongly associated with implementation levels achieved.

- Specific delivery moderators
- Staff engagement and training:

Staff engagement had a major impact of implementation levels. Resistance to additional demands (requiring planning and knowledge outside their normal remit) frequently inhibited staff, who clearly felt they already worked at or beyond capacity from becoming involved:

*‘this has been a very unhappy project for me I would have liked to have done an awful lot more but without buy in from the rest of the staff it’s been a bit of a lone ranger thing and it’s been very unsatisfactory.’* (Site C: IPB only)

While the study design explicitly contrasted implementation with/without KCS training and facilitation (IPB+: IPB only), it was anticipated that the IPB itself would form the basis for further in-house training and dissemination. However this was extremely varied, including for example: a whole team inset day, integration into existing staff meetings and self-directed learning. The content and coverage of training per se appeared less significant in determining implementation levels than the engagement of those involved, although these factors were clearly interrelated. Two successful strategies were:

i) identifying and training a project lead
  *‘D was interested, and has gone with it, and that being her area … I think that works best really, because … the breadth of areas is so great that people tend to have their own area…that they lead.’* (Site B: IPB+)
ii) wide staff involvement
  *‘we are a big staff team there is 70 odd of us, the community team that work … closely around these issues … they have all received the training.’* (Site C: IPB+)

b) Staff continuity:

While singling out individual staff to lead IPB implementation was an effective strategy which worked extremely well for many *‘essential*’ or *‘extended’* implementers, problems emerged when these individuals were relocated or left. This aspect of organisational change (frequently compounded by extremely limited or absent hand-over) affected 13 CCs:

*‘I didn’t work a lot with* [name] *prior to her going off sick so it’s just through lack of knowing anything about it really….I was aware in brief terms that there was a study going on … in terms of the IPB … I didn’t have a clue what that actually was.’* (Site D: IPB only)

CCs without a consistent project lead were less likely to achieve high implementation levels, although two overcame this by delegating implementation to outside agents. KCS facilitation also clearly provided continuity and information which helped to mitigate this effect.

c) Adaptability and flexibility:

Adaptability and flexibility in terms of approach used (when, how and by whom) to deliver the IPB were key attributes of successful delivery and required in-depth knowledge of the CC families, personal experience and creativity:

‘*For our families it’s about hearing a story … you know that they can sort of compare to so … it sparks a bit of imagination.’* (Site D: IPB+)

*‘I think it’s valuable adding things of your own that you think about … if there is a life lesson that you have learned along the way.’* (Site C: IPB+)

Factors which supported delivery were an opportunistic varied delivery style, targeting all-comers, including fun and/or children-friendly components, relating content to parents’ own experience, regular change in materials and activities and/or repetition of key facts. Additional pictorial, visual or role play elements, involvement of other agencies and researching and presenting local fire-related injury statistics were strategies which also helped.

*‘I think a condition of delivering the messages that you are just 100% honest and you don’t skirt about the subject …* (bring in) *something that’s happened in the community … where everybody knows about it … something that they can talk about or can relate to.’* (Site A: IPB+)

‘*A lot of our families have learning difficulties so … so for example the one about … children’s behaviour in fire prevention … was quite difficult to have it as a discussion with parents so we did it a little bit more visually, so we actually set up a role play area.’* (Site D: IPB+)

Implementation was more effective when integrated into existing sessions, although special events involving the Fire and Rescue Service (FRS) were also successful. Other positive strategies included ensuring parents could choose to opt in or out, involving children and acknowledging the difficulties many families lived with:

*‘you might have to tell them 20 times … to move their* (hair) *straighteners but you know one day you go back and they have actually been moved… it is work in progress … and I think with a lot of the families you have got to drip it in very slowly… some of them have got such major stuff going on.’* (Site C: IPB+)

Inability to adapt delivery content or processes was a major inhibitor for some:

*‘I think because in our heads we decided we were going to run them as workshops and so then when it didn’t come about it was kind of like oh well we can’t do it then.’* (Site C: IPB only)

However, for three CCs, keeping it simple, structured, to script and discrete was a factor in ensuring limited implementation where otherwise it might have failed.

*‘You know time wasn’t on my side, but we just decided to do at least two sessions and try … and follow the lesson plan.’* (Site A: IPB+)

d) Other agency support:

Other agency support (in particular FRS) also affected implementation levels but differed to the facilitation offered by KCS researchers (reported in section f). FRS involvement had a positive effect on parental engagement, often through involving children. FRS staff represented knowledgeable, instantly recognisable visitors, with equipment and practical displays adding interactive activities and interest to what could otherwise have appeared a dry topic. They helped to maintain consistency and supplement knowledge which improved CC staff confidence. They sometimes provided instant follow-up on identified safety issues. Some CCs were unable to access FRS support due to last minute emergency call-out and changes in FRS remit; this had an effect on implementation:

*‘We were hoping actually to get the fire brigade to come in to one of our stay and plays but… they can’t justify coming in which is a real shame…because of their financial cutbacks.’* (Site C: IPB+)

*‘I guess we would have used it more* (the IPB*) if we’d have … if the fire engines could have been here.’* (Site D: IPB only)

The six CCs in the minimal/non-implementation groups all had ‘normal FRS activity’ during the study period (fire engine or community fire safety officer visit). Health visitors (HV) (public health nurses employed by the NHS who frequently work alongside CC staff providing support to their families) were involved in implementation in three CCs. In one case, a HV student successfully completed ‘essential’ implementation independently. Two CCs recorded unsuccessful attempts in engaging HV support but acknowledged other demands on HV time:

*‘I don’t want to be critical of the health visiting team … it was going to be a joint effort and they were sort of well we haven’t got time and that was it so it was left in our court.’* (Site A: IPB+)

*‘They (HV) just haven’t got the time and the capacity again it’s frustrating… the health visitors don’t do half of what they used to do.’* (Site C: IPB+)

e) Conflicting targets or priorities:

While all CCs were expected to deliver parent education on a range of topics, a perceived lack of evidence to prioritise fire safety prevention (based on local directives and statistics) impeded some CCs implementation. Indeed this was the key factor in one CC’s inability to implement the IPB:

*‘I analysed the data for the area … and really drilled down to what the priorities were … low educational rates…a high rate of parents accessing support for mental health issues …there’s a high obesity rate … so immediately they were my priorities in terms of service delivery and … when I looked at any of the data regarding safety … there didn’t seem to be any.’* (Site D: IPB only)

**Figure 1:**
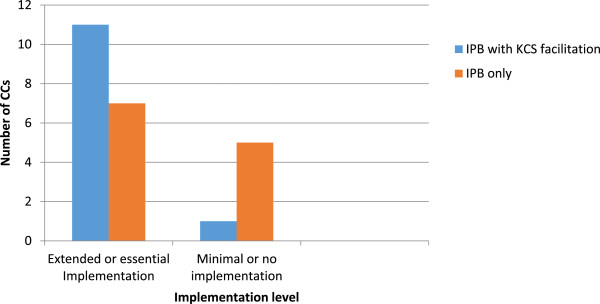
**The relationship between implementation level and KCS facilitation: number of children’s centres (N = 24) achieving extended/essential implementation and minimal/no implementation in the two intervention arms (IPB = and IPB only).**

f) Facilitation:
  KCS facilitation had a marked effect on level of implementation through enhancing ‘staff engagement’, ‘adaptability and flexibility’ and reducing potentially negative impacts of lack of ‘staff continuity’, ‘other agency support’ and ‘conflicting targets’. More CCs in the IPB+ arm achieved higher levels of implementation (11) than in the IPB only arm (7) and fewer achieved minimal or no implementation. Figure  1 demonstrates this relationship.

The initial training session was considered particularly useful in familiarising CC staff with the IPB and gave them the confidence to speak to parents about fire safety. Those in the IPB+ arm were also more likely (than IPB only CCs) to develop an implementation plan, share information about the IPB with other staff members and to use multiple delivery methods. On-going facilitation provided ideas, suggestions and encouragement (although some participants felt this could have been offered sooner. Perceived advantages of facilitation are evident in the IPB only and IPB+ participant quotes in Table 5.

**Table 5:** **IPB only and IPB+ participants’ comments on the advantages of KCS facilitation**

IPB only	IPB+
• *‘I think if someone had come in and … explained … what it was that you were doing and things then it probably would have… possibly would have made it so more got done.’* (Site C: IPB only)	• *‘The training at the start was … brilliant and it made it really … easy to follow the IPB and gave us the background knowledge that we needed to be able to speak to the parents about the things.’ (Site B: IPB+)*
	• *‘I think in terms of facilitating well the university has kick started us into it we wouldn’t have done it otherwise.’ (Site C: IPB+)*
	• *‘You’ve actually been very good at geeing me up to … you know because otherwise I have to admit … under the circumstances I think that’s exactly what we needed was the nagging … it gave us opportunities to … ask you anything.’ (Site D: IPB+)*
	• *Some felt facilitation was pivotal in enabling them to overcome the difficulties in engaging parents and of organisational change:*
	• *‘I think without it [facilitation] you wouldn’t get any results.’ (Site C: IPB+)*

### IPB complexity and effects on implementation in the CC context

The IPB was regarded positively by most CCs. Indeed 20 (83%) CCs felt the IPB was pitched ‘about’ or ‘just right’, only two felt it was too complex (two further minimal/non implementers did not comment). Most CCs found it straightforward, accessible and adaptable for different parent populations; it facilitated delivery of fire safety messages, aided confidence and provided "legitimate" knowledge. Nine CCs recorded parental safety behaviour change that ensued directly from delivering the IPB. The quotes in Table 6 illustrate these points.

**Table 6:** **Participant (IPB + & IPB only) comments on the IPB**

***IPB +***	• *‘I think it’s the materials … have been really helpful… they are easy to use and … to understand … they’re usable across different groups of parents like … teenage parents and with older parents. They were understandable and clear they get the message really pretty clearly.’ (Site B: IPB+)*
	• *‘I think the value of the material is that it’s fairly straightforward to use it’s all there together, bound in a book … we would not have been delivering anything around fire safety … without having an accessible tool like this.’ (Site C: IPB+)*
	• *‘We used the IPB and we had the fire safety little booklet as well so we had a lot of information, and so we came across as we knew what we were talking about.’ (Site B: IPB+)*
***IPB only***	• *‘The fact that the activity was there and there was … a session outline … how you could do this… so when you have got somebody who is … carrying two workloads … that kind of helped with the planning.’ (Site B: IPB only)*
	• *‘For us there was one …outstanding piece of work really that came from it …there was a real safety issue that was flagged up with one of the parents …she’d mentioned several things about wires being openly exposed in the walls … the fire station officers… made an appointment to go out that afternoon … and to put everything right for her.’ (Site A: IPB only)*
	• *‘I had a parent just last week … who had been to one… with the fire engine … she had someone come and check her house last week and you know they’ve done a safety plan.’ (Site D: IPB only)*

Only two CCs made negative comments about the IPB, both also made positive comments (the following quotes were made by the same participant):

*‘They are … not really geared up to being presented to the most … vulnerable … families that perhaps need that information its … a bit middle class.’* (Site A: IPB only)

*‘So it’s not that the messages are wrong I just think it’s about is having enough people to deliver it in an appropriate way.’* (Site A: IPB only)

Views about the IPB did not moderate implementation levels although ‘essential’ or ‘extended’ implementers were clearly more familiar with it and made more detailed comments. Eight (33%) specifically stated that the IPB itself had not been a factor which limited their implementation.

‘*If there was a problem … that was down to our families really, not … the IPB.’* (Site D: IPB+)

### What improvements can be made to the IPB?

Fifteen CCs found the IPB useful but suggested minor modifications (most frequently additional visual or simplified material and tailoring contents to specific parent groups):

*‘translations or making … it a bit less wordy perhaps because of the different languages that you have and parents with poorer literacy skills’ (Site C: IPB only)*

*‘I think as a set of information its brilliant. The session plans are great. I wonder if that might be worth perhaps doing almost like sound bites I suppose to whole sessions.’* (Site C: IPB only)

Table 7 lists improvements or additions to the IPB suggested by interview participants.

**Table 7:** **Suggested IPB improvements by number of CCs suggesting each improvement**

Improvement	No. CC
Simplification of content	7
Tailoring content to specific groups (different cultures, accommodation types, learning styles and abilities)	7
Increasing visual appeal	7
Including detachable resources for reproduction (or use during home visits)	6
Providing more interactive strategies (particularly those including activities for children)	4
Providing ‘sound bite’ materials for rapid delivery of key messages	3
Increasing local/parental relevance (through local fire related injury statistics and causes e.g. hair straighteners)	3

### Parental and staff response to successful IPB implementation

The previous sections demonstrate the circumstances affecting all CCs implementation and those that only affected some. However, when barriers to implementation were successfully overcome the IPB acted as a positive stimulant and parental and staff responses were overwhelmingly positive. Using the IPB raised awareness of the importance of fire safety, was enjoyable, prompted parental behaviour change and stimulated discussion in and beyond the CCs. One parent was reported as spontaneously leading an additional group and one sat through a repeat session. All CCs found it difficult to engage parents but once they were ‘*through the doors’* (Site B: IPB+) staff were frequently surprised by the depth of their engagement:

*‘I have been really surprised by the sort of quality of the discussion and reflection and I think it is simply because of going through that process of reflecting on the seriousness of fires the material that you are given.’* (Site C: IPB+)

*‘it’s difficult promoting it and … its difficult engaging families but once doing it, it’s surprising how much families do engage in it and fantastic to see that people are learning …without being involved in this study I wouldn’t have thought… about delivering fire safety training in our groups we might talk about a few of the issues but I wouldn’t have thought about systematically sitting down and thinking about the particular areas that the pack invites you to think about.’* (Site B: IPB+)

They felt there were clear benefits and hoped to continue to use the IPB in the future:

*‘We could see the benefit to the parents. We want to ensure that these* (IPB activities) *are kept on our agenda now …to include them in our sessional planning.’* (Site A: IPB only)

*‘They’re* (IPB activities) *just easy to use and people who came along, they got a lot out of it… we did evaluation and they scored it really highly and they said they had learnt something.’* (Site B: IPB+)

*‘Yeah I mean every parent that has come in and participated in a session has liked it and have gone away saying oh! We’ll think about doing a fire plan, we’ll check-up our alarms.’* (Site B: IPB+)

## Discussion

The IPB showed considerable promise as a tool to aid implementation of key fire safety messages in the CC context. Three-quarters of the CCs achieved high levels of implementation fidelity through ‘essential’ or ‘extended’ delivery. They reported positive parental response to the IPB material and most found it relevant, adaptable, simple and accessible. The IPB provided access to legitimate up-to-date evidence and activities to aid the planning and delivery of consistent messages. It stimulated staff enthusiasm and confidence and inspired future fire safety activities. Some minor improvements to the IPB were suggested but the IPB itself was not regarded as a factor that impeded implementation; it offered a dependable resource [2] for delivery to a range of parent groups and in a range of settings. However, higher levels of implementation were frequently achieved through abandoning trial requirements to deliver to recruited parents (this is likely to impact on the trial outcome). While this qualitative analysis did not seek to measure parental behaviour change, a number of changes were attributed to IPB delivery.

Analysis revealed a range of moderators that impacted on IPB implementation levels; these exerted either a ‘universal’ or ‘specific’ effect. Organisational change and difficult to engage populations were universal factors that affected all participating CCs’ implementation. Participants described numerous physical, environmental, educational and socio-economic circumstances affecting parental engagement and seemingly constant organisational, policy and funding changes; these illustrate the challenges of delivering public health initiatives in this context [9, 15, 20]. Effects of these ‘universal’ moderators though pronounced, were by nature unpredictable and difficult to control and as such may be hard to change. While CC staff used many strategies to overcome these issues, they impacted universally on IPB implementation and were likely to have influenced trial primary outcome measures.

Six specific moderators were directly associated with implementation levels achieved, namely: staff engagement and training, staff continuity, adaptability and flexibility, other agency support, conflicting health targets and KCS facilitation. These delivery factors had an important effect and were pivotal in determining minimal or non-implementation (but were unrelated to the IPB itself). This confirms the impact of ‘deliverer constraints’ on CC delivery of health promotion messages [8, 15]. While the appointment of a dedicated project lead was successful in aiding implementation, it was particularly vulnerable to staff change (54% CCs in the two intervention arms lost their project lead) and resulted in the remaining practitioners lacking basic information to support successful implementation. CCs were universally affected by changes in their own organisation; effects of other agency (FRS, HV) re-organisation and constraints were experienced by some more than others. While CC staff demonstrated remarkable commitment, resourcefulness and creativity, they could not always overcome these significant barriers despite advantages provided by the IPB.

However, combining the IPB with external (KCS) facilitation proved extremely successful in supporting implementation and overcoming ‘specific’ implementation moderators. Indeed while 58% of IPB only CCs achieved ‘high implementation fidelity’ [18] through ‘essential ‘or ‘extended’ implementation, this rose to 92% in the IPB+ arm. Analysis of the data suggested that facilitation could enhance ‘staff engagement’ and ‘adaptability and flexibility’ of delivery and mitigate barriers caused by staff change, lack of other agency support and conflicting priorities. While a ‘trusted deliverer’ improves uptake of information [8], informed outsiders and external support are also clearly important as additional sources of interest, inspiration, continuity and knowledge. The facilitation provided by trial researchers was designed to mimic that which ‘injury prevention co-ordinators’ might provide [21]. It remains unclear exactly how facilitation might look or cost outside the trial setting; this requires further exploration.

This study suggests that collaboration between research and practice can promote the design of simple, flexible interventions which are responsive to their target setting and audience [1, 2]. It also confirms the importance of understanding relationships between evidence, context and facilitation in promoting successful implementation [5, 22]. This study gives rise to a number of recommendations for policy, practice and research (see Table 8).

**Table 8:** **Key findings and implications for policy, practice and research**

**Key findings:**	
1.	The considerable challenges of engaging with this audience and of frequent organisational change should not be underestimated
2.	The IPB design methodology produced a tool aiding CC staff to deliver fire safety messages which:
	• was accessible to a broad range of staff
	• was adaptable to different audiences and simple to use
	• was a source of useful legitimate evidence
	• motivated staff to have a go
	• inspired future fire safety activity
	• generated parental discussion and interest
	• and initiated parental behaviour change
3.	While the IPB alone could not overcome all the challenges to implementation in this context combining it with external facilitation was extremely successful in improving:
	• Staff engagement
	• Adaptability and flexibility and in mitigating effects of:
	• Staff changes
	• Lack of other agency support
	• Conflicting priorities and targets
**Implications for policy practice and research:**	
1.	Future children’s centre injury prevention interventions need to address the difficulties posed by organisational change and audience engagement.
2.	Their design should ensure conditions for successful implementation are promoted through incorporating contextual knowledge and facilitation.
3.	They should provide supporting evidence of local need and be accompanied by policy directives to enable CC staff to prioritise them.
4.	Facilitation should include:
	• Internal facilitation: A named member of staff who is responsible for leading this strand of work and monitoring the impact.
	• External facilitation: possibly drawing on the expertise of local injury prevention teams; especially the local FRS to answer queries, share concerns and raise confidence levels.
	• Consistent involvement of external agencies including local Fire and Rescue Services is also important.
5.	IPBs are a potentially promising intervention for use by children’s centres, but they require evaluation in terms of safety behaviours and injury outcomes.
6.	Possibilities for expanding the methodology for IPB development to other public health areas should be explored through further research
7.	Further changes in CCs organisation, funding, and priorities should consider the impact this has on effective delivery of services.

Participant accounts identified minor modifications to the IPB and its delivery to improve future implementation. Key areas were simplification, increased pictorial content, child friendly activities, improved accessibility to specific parent populations, introduction of detachable resources for reproduction and ‘sound bites’ for rapid dissemination. This supports evidence that simple interventions directed at behaviour change are more successful. [1, 2, 8] and provides useful information for future IPB and public health intervention development.

### Study strengths and limitations

The views of service providers were incorporated into the IPB design process. However, parental involvement in study design and implementation might have added additional insight into what works for the target group. This study benefitted from the perspectives of CC staff working in a range of urban/rural areas serving varied populations in terms of size, organisation, funding, and socio-economic/ethnic mix. This enabled us to test IPB implementation in different settings among varied populations, and to explore the experiences of a range of staff. While our findings cannot be generalised to all CCs, this study provided a range of perspectives which are likely to be representative of many UK CCs. Conduct of the interviews, facilitation and analysis by research staff with varied academic and professional backgrounds enhanced the transferability and credibility of our findings by ensuring a range of perspectives and interpretations were considered. KCS researchers participated in both intervention facilitation (IPB+) and 12 month interviews. While this may have influenced participant accounts, it was mitigated by these researchers’ interview experience and use of a structured topic guide. Furthermore, most participants (including those in the IPB only arm and those who had not been in post from the outset) had little prior acquaintance with their interviewer. Trial processes created additional layers of complexity which would not exist in the real-world; this had a clear effect enabling some CCs to achieve minimal levels of implementation and limiting more extensive implementation in others. Researcher knowledge of CC study arm and site may have influenced analysis although this was mitigated by contrasting perspectives of one researcher new to the study (KB) and one who had been involved from the start (TG). Use of the Implementation Fidelity Framework [18], both prospectively to inform interview schedule design and retrospectively within the analysis, aided systematic measurement of implementation fidelity. This is important in terms of study replication, evidence-based practice and future development of the IPB [18]. This qualitative study explored parental perspectives through the eyes of CC staff only and did not seek to establish the relationship between IPB implementation and parental fire safety practices (which will be measured by the quantitative data within the trial). Description of this study and methodology have been subject to review according to the RATS guidelines for Qualitative research to aid reader critical appraisal (see Additional file 1) [23].

## Conclusion

The IPB has significant potential to improve implementation of evidence-based fire safety messages and overcome known, considerable, and often unpredictable challenges in the CC context (especially when combined with external facilitation). The seven step design process adapted by Brussoni et al. [1] resulted in a simple, informative, accessible and comprehensive guide with relevance and practical utility in this context. There are economic and resource implications of such a lengthy design process but these may be offset against the individual, NHS and societal costs of injury. This model combining the art and science of injury prevention has potential to enhance implementation of public health interventions and to support frontline staff charged with their delivery; it could be used in a range of settings and other public health areas. Similarities between this approach and developments in ‘public health detailing’ or ‘academic detailing’ could be explored [24].

The Implementation Fidelity Framework [18] has potential to improve understanding of why or how an intervention works and its potential contribution to outcomes. Simultaneous use of framework analysis [19] permits systematic exploration of other factors affecting implementation; in this case revealing how the relationship between implementation moderators and intended outcomes may not always be linear or predictable. Those with more straightforward impact on implementation levels may be more amenable to change. These findings and the methodology used in IPB design and evaluation may be of future significance for public health interventions and implementation research.

## Appendix 1 Example interview questions

1. For those fire safety messages you promoted, I am going to ask you for the reasons why you could/could not promote them as often or as long as planned.
   For fire safety message (a), how long did this take? Was it by a formal or informal session? Please describe what you did and/or who was involved.
2. Overall, you agreed/disagreed that parents were fully engaged in the fire safety messages and advice provided by your Centre.
  – Why was this?
  – Can you give us any examples about how parents were engaged?
3. I would like to talk about the level of the fire safety messages.
   How complex did you feel the fire safety messages were? Did you feel they were too complex, about right or too straightforward?
   Can you tell me why?
4. Can you identify the barriers and facilitators that affected the way that you promoted the fire safety messages?
   You said there were some factors that affected the way in which the fire safety messages were promoted.
   What were these?
   Did these have any effect on how your Centre promoted the fire safety messages?

## Electronic supplementary material

Additional file 1: **Qualitative research review guidelines – RATS applied to article submission.** (DOCX 21 KB)

## Abbreviations

KCS: Keeping children safe programme
CC(s): Children’s centre(s)
HV: Health visitor
IPB: Injury prevention briefing
IPB+: IPB delivery supplemented by initial training and on-going facilitation
IPB only: IPB mailed out no facilitation provided
FRS: Fire and rescue service
NIHR: National institute of health research
UK: United Kingdom
NHS: UK national health service
Site A: B, C, D: used to anonymously identify the four UK study sites (Bristol, Newcastle, Norwich and Nottingham).
