# Supplementary material for: Implementing an Injury Prevention Briefing to aid delivery of key fire safety messages in UK children’s centres: qualitative study nested within a multi-centre randomised controlled trial
Source: BMC Public Health. 2014 Dec 10;14:1256. doi: 10.1186/1471-2458-14-1256 (PMC4295482; doi:10.1186/1471-2458-14-1256)
Supplement: Supplementary file 1 — Additional file 1: Qualitative research review guidelines – RATS applied to article submission. (DOCX 21 KB) [file 12889_2014_7376_MOESM1_ESM.docx]

# **Qualitative research review guidelines – RATS** **applied to article submission:**

# **Title: Injury prevention briefings: a strategy to aid implementation of community public health interventions among at risk populations? Qualitative study nested within a multi-centre randomised controlled trial**

# **Authors’ names:**

Kate Beckett^1^, Trudy Goodenough^2^, Toity Deave^3^, Sally Jaeckle^4^, Lisa McDaid^5^, Penny Benford^6^, Mike Hayes^7^, Elizabeth Towner^8^, Denise Kendrick^9^ and the Keeping Children Safe Programme study group

| **Guideline** | **How Addressed** | **Page No.** |
| --- | --- | --- |
| **R Relevance of study question**  Is the research question interesting?  Is the research question relevant to clinical practice, public health, or policy? | We have explicitly stated the research question and made links to existing empirical, theory and policy knowledge base | 3-5 |
| **A Appropriateness of qualitative method**  Is qualitative methodology the best approach for the study aims? | The study design is described and justified; readers are also directed to the trial published protocol for more detail.  Interviews are specified as a means to explore Implementation fidelity by the framework authors (Carroll et al; 2006) | 6-8 |
| **T Transparency of procedures**  *Sampling*  Are the participants selected the most appropriate to provide access to the type of knowledge sought by the study?  Is the sampling strategy appropriate? | The criteria for selecting the study sample are already published in detail in the trial protocol which is referenced. We describe which of the trial CCs participated in the interviews. | 4,6 |
| *Recruitment*  Was recruitment conducted using appropriate methods?  Is the sampling strategy appropriate?  Could there be selection bias? | The criteria for recruitment are already published in detail in the trial protocol which is referenced (file uploaded with submission). | 4,6 |
| *Data collection*  Was collection of data systematic and comprehensive?  Are characteristics of the study group and setting clear?  Why and when data collection was stopped, and is this reasonable? | Data collection is described in detail; setting and study group characteristics are also described in the trial protocol. We have attached example questions in an appendix. Data collection adhered to trial protocol. | 5-8 |
| *Role of researchers*  Is the researcher(s) appropriate? How might they bias (good and bad) the conduct of the study and results? | The role of researchers in IPB facilitation and interviews is described. Their positions and organisations are provided. Overcoming possible bias in interpretation is discussed in ‘study limitations’. | 1,5,28 |
| *Ethics*  Was informed consent sought and granted?  Were participants’ anonymity and confidentiality ensured?  Was approval from an appropriate ethics committee received? | Informed written consent was sought and granted and anonymity ensured. Ethics approval was granted and evidenced in an ethics statement. | 8 |
| **S Soundness of interpretive approach**  *Analysis*  Is the type of analysis appropriate for the type of study?   - *thematic:* exploratory, descriptive, hypothesis generating - *framework:* e.g., policy - *constant comparison/grounded theory:* theory generating, analytical - Are the interpretations clearly presented and adequately supported by the evidence? | We have justified our use of a qualitative methodology and framework analysis.  We have aimed to present our interpretation clearly and support it with evidence | 5,7,8  8-30 |
| Are quotes used and are these appropriate and effective? | Quotes have been selected to represent themes in the data | 8-25  And in boxes |
| Was trustworthiness/reliability of the data and interpretations checked? | The data were checked by cycles of primary coding and intercoder reliability testing by other KCS researchers | 6-8,28 |
| *Discussion and presentation*  Are findings sufficiently grounded in a theoretical or conceptual framework?  Is adequate account taken of previous knowledge and how the findings add?  Are the limitations thoughtfully considered?  Is the manuscript well written and accessible? | Our findings are grounded within the Carroll et al (2006) ‘Implementation fidelity framework’ and within the extensive literature on translating evidence into practice. We aim to have identified all possible limitations. We aimed to write in an accessible style and to make this manuscript easy to read and comprehend. | 5-7, 10, 25-30 |
| **Are red flags present? These are common features of ill-conceived or poorly executed qualitative studies, are a cause for concern, and must be viewed critically. They might be fatal flaws, or they may result from lack of detail or clarity.** | We are not aware of the presence of any ‘red flags’ |  |
